# The Effect of Low-Temperature Crystallization of Fish Oil on the Chemical Composition, Fatty Acid Profile, and Functional Properties of Cow’s Milk

**DOI:** 10.3390/ani10101834

**Published:** 2020-10-09

**Authors:** Robert Bodkowski, Katarzyna Czyż, Ewa Sokoła-Wysoczańska, Marzena Janczak, Paulina Cholewińska, Anna Wyrostek

**Affiliations:** 1 Institute of Animal Breeding, Wrocław University of Environmental and Life Sciences, Chełmońskiego 38c, 51-630 Wrocław, Poland; katarzyna.czyz@upwr.edu.pl (K.C.); marzena.janczak@upwr.edu.pl (M.J.); paulina.cholewinska@upwr.edu.pl (P.C.); anna.wyrostek@upwr.edu.pl (A.W.); 2 The Lumina Cordis Foundation, Szymanowskiego 2a, 51-609 Wrocław, Poland; sokola@libero.it

**Keywords:** milk, fish oil enriched with *n*-3 FAs, DHA, EPA and CLA, desaturase index, atherogenic and thrombogenic indices

## Abstract

**Simple Summary:**

Nowadays, both consumers and producers are paying increasing attention to the health-promoting properties of food (functional food). Wide and multi-directional pro-healthy effects are shown by long-chain unsaturated omega-3 fatty acids (mainly eicosapentaenoic (EPA), and docosahexaenoic (DHA) ones) and conjugated linoleic acid dienes (CLA). On the other hand, saturated fatty acids (SFAs), particularly medium-chain ones, have adverse atherogenic and thrombogenic effects. The supplementation of dairy cows’ rations with fish oil subjected to low-temperature crystallization (LTC-FO) enriched with long-chain polyunsaturated fatty acids reduced milk fat yield and concentration and profitably modified the fatty acid profile. The level of SFAs, especially short- and medium-chain ones, in milk fat decreased. In turn, the content of polyunsaturated fatty acids (PUFAs), including biologically active ones like eicosapentaenoic (EPA), docosahexaenoic (DHA), CLA, and trans-vaccenic acid (TVA), increased. In conclusion, LTC-FO could be used to modify the nutritional and health value of milk.

**Abstract:**

The study aimed to investigate the effect of supplementation of fish oil after the process of low-temperature crystallization (LTC-FO) enriched with long-chain polyunsaturated fatty acids (LC-PUFAs) on cow milk parameters. The experiment was carried out on 24 Polish Holstein Friesian cows. For 4 weeks, experimental (EXP) group animals (*n* = 12) were fed LTC-FO (1% of dry matter). Milk was collected two times: on days 14 and 30. LTC-FO supplementation decreased milk fat yield and concentration (*p* < 0.01). Higher levels of polyunsaturated fatty acids (PUFAs), including these with beneficial biological properties, i.e., eicosapentaenoic (EPA), docosahexaenoic (DHA), docosapentaenoic (DPA), CLA, alpha-linolenic acid (ALA), and TVA (*p* < 0.01), and lower levels of SFAs, especially short- (*p* < 0.01) and medium-chain ones (*p* < 0.05, *p* < 0.01), were found in the EXP group. The addition of LTC-FO reduced the value of atherogenic and thrombogenic indices as well as SFA/UFA and *n*-6/*n*-3 ratios and increased the content of *n*-3 PUFA and functional fatty acids (*p* < 0.01). The addition of LTC-FO also increased the delta-9 desaturase index for CLA/TVA and decreased it for pairs C14:1/C14:0 and C16:1/C16:0 (*p* < 0.05, *p* < 0.01).

## 1. Introduction

Currently, agricultural research is aiming not only for an improvement in the yield and production outcomes but also in the nutritional value of food products. An example of a sector that has undertaken attempts to modify the fatty acid (FA) profile of milk fat, especially FAs beneficial for human health, is the dairy production sector [1,2,3]. Bearing in mind the potential long-term benefits for human health, there is an interest in establishing sustainable nutritional strategies to reduce the content of saturated fatty acids (SFAs) while concurrently increasing the levels of unsaturated ones (UFAs) [4].

In scientific and medical fields, omega-3 (*n*-3, ω-3) FAs exhibit multidirectional effects in human organisms [5]. Although a number of publications on the importance of *n*-3 polyunsaturated fatty acids (PUFA) in human health and nutrition (preventive, curative effect of their supplements) are available, a growing interest is still observed. Health benefits of *n*-3 FAs have been reported in the case of a number of diseases, including cardiovascular diseases, hypertension, atherosclerosis, diabetes, cancer, arthritis, asthma, depression, skin disorders, chronic inflammatory, autoimmune, and neurological ones; they also can enhance lipid metabolism regulation and support central nervous system functioning [5,6,7]. For this reason, various organizations have established dietary recommendations and guidelines for the intake of *n*-3 FAs, particularly eicosapentaenoic acid (EPA, C20:5n-3) and docosahexaenoic acid (DHA, C22:6n-3) [8,9]. Owing to low fish consumption in many European countries and USA, the average consumption of *n*-3 long-chain PUFA (LC-PUFA) is below the recommended level [8].

The main energy component of cow’s milk is fat 3.8–5.9% [10,11], whose significant nutritional value, high digestibility, and biological properties of FAs are the reason for its important role in the human diet. Less than 15% of the total fat in the human diet and nearly 25% of the total saturated fat originate from milk and dairy products in western diets [12]. The dominant group of FAs of cow’s milk are SFAs (> 64% total FAs) [1,10], mainly medium-chain fatty acids (MCFAs), particularly lauric (C12:0), myristic (C14:0), and palmitic ones (C16:0), which may contribute to an increased risk of cardiometabolic or cardiovascular diseases [13]. UFAs constitute around 30–35% of cow’s milk fat, including PUFAs, at approximately 2.8–3.7%, mainly linoleic acid (LA, C18:2n-6) and α-linolenic acid (ALA, C18:3n-3) [1,10]. In turn, *n*-3 LC-PUFA (> C20), including EPA and DHA, are present in small amounts or below the detection threshold in cow’s milk fat (EPA 0.03–0.067 g/100 g and DHA < 0.002–0.03 g/100 g of total milk FAs) [1,2,14].

Milk and its products are traditionally some of the main components of the human diet, but they are concurrently one of the worst sources of *n*-3 PUFAs. The concentration of these FAs, including EPA and DHA in cow’s milk, can be elevated by the application of oilseeds or plant oils [15,16], fish oil [14,17], fish and plant oils used concomitantly [1,18], as well as marine or micro-algae [19,20]. The supplementation of fish oil also causes an increase in the content of conjugated linoleic acid (CLA), exhibiting very beneficial and multidirectional pro-health effects [21], as well as trans-vaccenic acid (TVA, C18:1) in milk fat [22].

Most of the papers on the influence of fish oil application in ruminant nutrition on the chemical composition of milk and the profile of FAs concerns different doses, forms, and sources of origin. However, there is no literature available on the possibility of using fish oils with an increased n-3 PUFA content. The objective of our study was to examine the effect of the addition of fish oil after the process of low-temperature crystallization (LTC-FO) enriched with long-chain polyunsaturated fatty acids (LC-PUFA) in cows’ diet on (1) milk yield as well as yield and concentration of milk ingredients, (2) composition of individual FAs, (3) content of FA groups with different saturation degrees and carbon chain lengths, total CLA, EPA+DHA, and functional FAs, SFA/UFA and *n*-6/*n*-3 ratios, activity of delta-9 desaturase for substrate/product, as well as the values of atherogenic (AI) and thrombogenic (TI) indices. The present experiment tested the hypotheses that LTC-FO may (1) lower milk fat, (2) increase the concentration of EPA, DHA, CLA, and TVA in milk fat, (3) increase the content of PUFA and decrease the content of SFA as well as the value of the AI index, (4) that the effects depend on the time period of supplementation.

## 2. Materials and Methods

### 2.1. Process of Low-Temperature Crystallization of Fish Oil and Feed Additive Elaboration

Raw fish oil was obtained during the production of fish meal, as a result of boiling fish waste (mainly herring and sprat from the Baltic Sea) and then pressing the resulting pulp. The resulting post-production waters (temperature > 90 °C) were directed successively to a clarifying and separating centrifuge to separate the crude fish oil. In order to reduce the content of dioxins and dioxin-like compounds, fish oil was subjected to a purification process [23].

The concentration of *n*-3 FAs in fish oil, mainly EPA and DHA, was increased by the process of low-temperature crystallization (LTC) [24], according to a modified version of the method elaborated by Bodkowski et al. [25]. The process of FA transesterification was carried out using 2 M methanol solution of potassium hydroxide. First, 2500 mL of acetone (Avantor Performance Materials Poland Inc., Gliwice, Poland) was added to 1000 mL of fatty acid methyl esters (FAME) of fish oil, and it was mixed for 2 min (300 rpm) using a magnetic stirrer (Ikamag EDA 9, IKA^®^ Poland Ltd., Warsaw, Poland) and then was placed in the freezer (Thermo Electron Corporation, Waltham, MA, USA) at −70 °C for 18 h. After the freezing process, the crystallized fraction was separated from the liquid part by means of a laboratory kit for vacuum filtration using a Buchner funnel (Laboport, KNF Neuberger, Inc, Trenton, NJ, USA). In the last stage, acetone was evaporated (40 °C, vacuum 200 mbar) from the liquid fraction using a rotary vacuum evaporator (Laborota 4011, Heidolph Instruments GmbH & Co. KG Vertrieb Labortechnik, Schwabach, Germany) and it was used again in the next cycle.

To protect UFA in fish oil after the process of low-temperature crystallization (LTC-FO) against oxidation, α-tocopherol dissolved in methanol (all reagents from Avantor Performance Materials Poland Inc., Gliwice, Poland) was added at 200 mg/100 g [26]. Up to the time of application tests on cows (max. 2 weeks), LTC-FO was stored in refrigerated conditions (approx. 4 °C) in closed dark bottles.

Chromatographic analysis of FAME was performed using a gas chromatograph (Agilent Technologies, Santa Clara, CA, USA) equipped with a flame ionization detector (FID) and SP-2560 capillary GC column (100 m length × 0.25 mm inner diameter (i.d.), d_f_ = 0.20 μm; Supelco, Bellefonte, PA, USA). The temperatures of the injector and detector were 250 and 260 °C, respectively. Nitrogen was used as the makeup gas, while helium was the carrier gas. The flow rate of the carrier gas was 1.1 mL/min. The oven program was as follows: initial temperature 140 °C (2 min), increase 2 °C/min up to 225 °C, isotherm for 10 min, increase 4 °C/min up to 240 °C, isotherm for 10 min. Two microliters of the sample was injected in split mode (100:1). Particular FAs were identified by comparison with FAME standards (GLC #47885, #47571, #H4515, #20290-75-9, #17269, #10417-94-4, #D2659 Sigma-Aldrich, Chemie GmbH, Schnelldorf, Germany). The process of transesterification and chromatographic analyses were carried out according to the procedures ISO [27] and AOCS [28]. The process of low-temperature crystallization of fish oil (FO) and chromatographic analysis of FO and LTC-FO were conducted at the Department of Food and Environmental Chemistry, National Marine Fisheries Research Institute in Gdynia, Poland (6 analyses for FO and LTC-FO, from different batches of oil).

### 2.2. Animals and Treatments

The experiment was conducted on 24 Polish Holstein Friesian multiparous cows of the red-white variety with body weight of 602 ± 14.8 kg, at 90 ± 23 d of lactation, and milk production of 31.2 ± 3.12 kg/d, divided into 2 groups (*n* = 12/group). The animals were chosen from the herd of 320 cows, the body weight and milk yield of which were comparable, and were randomly allocated to the groups (CTRL—control, EXP—experimental; *n* = 12 head/group). The cows were individually housed in a stall system. They were fed total mixed ration (TMR) based on corn and grass silage, with ad libitum access to fresh water. Animals from the EXP group were supplemented with fish oil after the process of low-temperature crystallization (LTC-FO). The amount of supplement was 1% of dry matter (DM) and it was applied on the mineral carrier Humokarbowit. Cows from the CTRL group were supplemented with Humokarbowit in an analogous amount (Table 1). The cows were handled according to the regulation of the Polish Council on Animal Care, and all procedures were approved by the 2nd Local Ethical Committee for Experiments on Animals in Wrocław, Poland (No. 61/2009).

The feed doses for cows were established according to the INRA system [29] on the basis of analyses of particular components, i.e., dry matter, crude protein, crude fat, and crude fiber, according to procedures by AOAC [28]. The methods of Van Soest et al. [30] were used in the analyses of neutral detergent fiber(NDF) and acid detergent fiber (ADF). Samples of TMR were pooled 3 times over experiment (at the start and after 10 and 20 days). In order to determine the intake of dry matter (DMI), all refusals were collected and weighed every day.

LTC-FO was nozzle-sprayed onto Humokarbowit in amount of 20% LTC-FO/kg of the carrier. The procedure was performed by the Tronina company (PHW Tronina, Raków, Poland).

### 2.3. Design of the Experiment, Analyses, and Sample Collection

Before the beginning of the experiment, all animals received for 14 days the same CTRL diet (adaptation period). The length of the study was one month (30 days). The supplements were administered with TMR in the morning (05:00 h). The cows were milked twice a day (at 06:00 and 16:00 h), and individual milk samples (100 mL) were collected after 14 and 30 days of experiment from the morning milking. The samples were cooled down (4 °C) and analyzed within 4 h after the sampling. Basic parameters of milk, i.e., dry matter, fat, protein, and lactose contents, were determined using Infrared 150 apparatus (Bentley Instruments Inc., Chaska, MN, USA), while hygienic status of the milk was determined based on somatic cell count (SCC) using Somacount 150 apparatus (Bentley Instruments Inc. Chaska, MN, USA). The analyses were performed at the Laboratory of Milk Assessment and Analysis in the Institute of Animal Breeding, Wrocław University of Environmental and Life Sciences (Poland).

Milk fat for chromatographic analysis (1 g) was obtained as a result of milk centrifugation at 4000× *g*, 4 °C for 15 min (MPW 260RH apparatus, Med. Instruments, Warsaw, Poland). The extraction of lipids was carried out in accordance with the modified procedure by Folch (chloroform and methanol in a ratio of 2:1 by volume), while the methylation process was conducted using 2 M KOH in methanol [1].

Chromatographic analyses were carried out in the Laboratory of Chromatography and Meat Analysis, Institute of Animal Breeding, Wrocław University of Environmental and Life Sciences (Poland) using gas chromatograph (Agilent Technologies 7890A) with FID detector and HP-88 capillary GC column (100 m length × 0.25 mm i.d., d_f_ = 0.20 μm; Agilent Technologies, Santa Clara, CA, USA). A detailed description of conditions for conducting analyzes is reported elsewhere [3]. The resulting peaks were identified by comparing the retention times with the standards (GLC #47885, #47791, #46903, #46904, #46905, #43959, #O5632, #D5679 Sigma-Aldrich Chemie GmbH, Schnelldorf, Germany, #20-1823-7, #20-1826-7, Larodan Fine Chemicals AB Products, Malmö, Sweden).

### 2.4. Statistical Analysis

The normality of all variables was tested using the Shapiro–Wilk test and when needed they were transformed into logarithm form in order to normalize frequency distribution.

The results of the experiment were analyzed using the procedure of the SAS (version 9.0, SAS Institute Inc., Cary, NC, USA). The differences between FA content in raw fish oil and after the process of low-temperature crystallization were established using Student’s *t*-distribution test at significance level of *p* < 0.05. Milk chemical composition and its fatty acid profile were calculated using two-way ANOVA based on a linear model that included the effect of diet (D), time (T), and their interactions (D × T).

The differences in ANOVA were established using Tukey’s test at significance level of *p* < 0.05.

## 3. Results

### 3.1. Process of Low-Temperature Crystallization of Fish Oil

The FA composition of raw sprat-herring oil (FO) and after the process of low-temperature crystallization (LTC-FO) are summarized in Table 2 (only FAs > 1 g/100 g of raw FO). To provide an overview of the results, FAs are grouped as saturated fatty acids (SFAs), monounsaturated fatty acids (MUFAs), and polyunsaturated fatty acids (PUFAs).

Raw fish oil contains a high level of UFAs (PUFAs + MUFAs > SFAs) and uniquely high levels of *n*-3 PUFAs, including docosahexaenoic acid (C22:6, DHA) and eicosapentaenoic acid (C20:5, EPA). Palmitic acid (C16:0) was the major FA among the SFAs and oleic acid (C18:1, OA) among the MUFAs.

The process of low-temperature crystallization (LCT) of fish oil increased the content of UFAs by 25.1%, including PUFAs by 73.3%, compared to raw fish oil (*p* < 0.01). The largest increase was recorded for C22:6 (DHA), C20:2 (EDA), C20:5 (EPA), and C22:5 (DPA) (*p* < 0.01). The increase in UFA content was accompanied by a decrease in SFAs by 74.1%, including C16:0, C18:0, and C14:0 (*p* < 0.01), as well as MUFAs by 19.9%, including C16:1 and C17:1 (*p* < 0.05, *p* < 0.01).

### 3.2. Chemical Composition of Milk

As demonstrated in Table 3, the supplementation of elaborated feed additive which is fish oil after the process of low-temperature crystallization (LTC-FO) to TMR did not influence the milk production level of the cows. LTC-FO also had no effect on the yield and protein and lactose content of milk. As a result of LTC-FO supplementation after 14 and 30 days of the experiment, fat yield decreased by 25.9% and 22.5% (*p* < 0.01), and its concentration in milk by 27.3% and 25.8% (*p* < 0.01), respectively.

### 3.3. Fatty Acid Profile of Milk

The addition of LTC-FO modified the FA profiles of milk fat (Table 4).

As a result of supplementation with LTC-FO in the group of short-chain fatty acids (SCFAs), we observed decreases in the content of butyric acid (C4:0) by 17.3% and 23.9%, caproic acid (C6:0) by 25.8% and 21.9%, caprylic acid (C8:0) by 22.6% and 38.8%, and capric acid (C10:0) by 27.7% and 37% after 14 and 30 days of the experiment, respectively, compared to the CTRL (*p* < 0.01). In turn, in the group of medium-chain fatty acids (SCFAs), the content of lauric acid (C12:0) decreased by 21% and 16.5%, miristic acid (C14:0) by 7.8% and 7.6%, and palmitic acid (C16:0) by 6.9% and 6.9%, respectively (*p* < 0.05, *p* < 0.01). Except the diet, the changes in C12:0 content were also affected by the time of LTC-FO supplementation (*p* < 0.05). Furthermore, the content of stearic acid in milk fat (C18:0) decreased by 10.8% and 15.8%, and oleic acid (*cis-9* C18:1) by 12% and 7.7%, after 14 and 30 days of the experiment, due to the use of the developed additive, compared to the CTRL group (*p* < 0.05, *p* < 0.01).

In turn, supplementation of cows’ feed ration with LTC-FO resulted in an increase in milk fat of palmitoleic acid (*cis*-9 C16:1) by 117% and 113.4%, isomers C18:1 of configuration *trans*-9 by 73.5% and 74.5%, *trans*-10 by 128.6% and 142.6%, *cis*-11 by 142.1% and 142.5% and *trans*-11 (TVA) by 326.1% and 377.3%, isomers CLA of configuration *cis*-9,*trans*-11 by 267.4% and 310.2%, *trans*-9,*cis*-11 by 200% and 210%, *trans*-10,*cis*-11 by 33.3% and 28.6%, α-linoleic acid (all-*cis*-9,12,15 C18:3, ALA) by 35.8% and 79.5%, γ-linoleic acid (all-*cis*-6,9,12 C18:3, GLA) by 100% and 126.7%, and stearidonic acid (all-*cis*-6,9,12,15 C18:4, SDA) by 266.7% and 380%, after 14 and 30 days, respectively, compared to CTRL (*p* < 0.01). Moreover, the content of C18:1 *t*10, ALA and GLA (*p* < 0.01) as well as C18:2 isomers *c*9,*t*11 and *t*9,*c*11 (*p* < 0.05) was affected by the time of LTC-FO administration (14 and 30 d), while the diet x time interaction was found for C18:1 *t*10, ALA, GLA, and SDA (*p* < 0.05, *p* < 0.01). In the case of CLA, a decrease was only noted for isomer *trans*-11,*cis*-13 by 36.4 and 47.4% (*p* < 0.01) after 14 and 30 days of LTC-FO application. This isomer content was also influenced by supplementation time (*p* < 0.01), and, additionally, a diet x time interaction was demonstrated (*p* < 0.05).

As a result of chromatographic analyses, it was found that after both 14 and 30 days of LTC-FO application, the content of most long-chain fatty acids (C > 20) in milk fat increased statistically, including eicosanoic acid (*cis*-11 C20:1) by 339.7% and 657.7%, eicosadienoic acid (*cis*-11,14 C20:2, EDA) by 294.8% and 353.6%, dihomo-γ-linolenic acid (all-*cis*-8,11,14 C20:3, DGLA) by 81.1% and 107.3%, eicosapentaenoic acid (all-*cis*-5,8,11,14,17 C20:5, EPA) by 143.7% and 170.6%, docosapentaenoic acid (all-*cis*-7,10,13,16,19 C22:5, DPA) by 146.8% and 177.3%, and docosahexaenoic acid (all-*cis*-4,7,10,13,16,19 C22:6, DHA) by 247.6% and 347.4%, compared to the CTRL group (*p* < 0.01). Moreover, in the case of C20:1, DGLA, EPA, and DHA, the spectrum of changes depended on the time of LTC-FO administration (*p* < 0.05, *p* < 0.01), and a diet x time interaction was found for C20:1 (*p* < 0.01).

Analyzing changes in the content of the groups of FAs differing with the degree of saturation and the length of carbon chain during the whole experiment, the proportion of SFAs decreased by 9.2% and 12.7%, SCFAs by 23% and 30%, and MCFAs by 3.4% and 4.9%, respectively, after 14 and 30 days, in the EXP group compared to CTRL (*p* < 0.05, *p* < 0.01) (Table 5). In contrast to the control group, 14 and 30 days of LTC-FO supplementation in milk fat increased the content of UFAs by 19.2% and 27.4%, MUFAs by 14% and 22%, PUFAs by 55.6% and 63.5%, and LC-PUFAs by 120.7% and 157.1%. In addition, in the case of UFA, PUFA, and LC-PUFA, the changes were also influenced by the time of LTC-FO application (*p* < 0.05, *p* < 0.01), and in the case of LC-PUFA, the diet x time interaction was noted (*p* < 0.01).

As a result of LTC-FO supplementation, also the content of Σ CLA in milk fat increased by 257.3% and 292.5%, EPA + DHA by 200% and 260%, functional fatty acids (FFA) by 8.9% and 16.3%, and *n*-3 FAs by 55.9% and 107.9%, while the ratio of SFA/UFA decreased by 23.8% and 31.4% and *n*-6/*n*-3 by 32.3% and 52.5%, respectively, after 14 and 30 days of experiment, compared to CTRL (*p* < 0.05, *p* < 0.01). The content of Σ EPA + DHA, *n*-3 FAs, and *n*-6/*n*-3 ratio was also dependent on the time of LTC-FO application (*p* < 0.05, *p* < 0.01), and a diet x time interaction was found for *n*-3 and *n*-6/*n*-3 (*p* < 0.01).

The addition of LTC-FO also effected a decrease in the value of the atherogenic index (AI) of milk fat by 22% and 28.1%, as well as the thrombogenic one (TI) by 21.4% and 32.1%, after 14 and 30 days of the experiment (*p* < 0.01). The value of the delta-9 desaturase index for CLA/TVA was subject to a decrease (*p* < 0.05), while it increased for C16:1/C16 (*p* < 0.01) and C14:1/C14:0 (*p* < 0.05).

## 4. Discussion

### 4.1. Process of Low-Temperature Crystallization of Fish Oil and Elaborated Feed Additive

As observed in the present study, the distribution of fatty acids PUFA > SFA and high level of *n*-3 FAs, including EPA and DHA, of raw sprat-herring oil is confirmed by the results of other authors’ research [17,32,33].

The biological value of fish oils is primarily determined by the concentration of *n*-3 FAs. There are several techniques allowing us to increase the level of *n*-3 FAs, mainly EPA and DHA, e.g., molecular or fractional distillation, adsorption chromatography, supercritical fluid extraction, enzymatic splitting, urea complexation, or low-temperature crystallization, but only some of them may be applied in cost effective production on a large scale. In our research, in order to enrich fish oil in *n*-3 FAs, we used a non-complicated low-temperature crystallization (LTC) method that did not require specialized equipment. LTC was elaborated to separate certain triglycerides (TG), fatty acids, esters, and other lipids that are highly soluble in organic solvents at temperatures above 0 °C but are poorly soluble at temperatures below 0 °C. Fat solubility in organic solvents decreases with an increase in mean molecular weight and increases with an increase in unsaturation degree [34]. It was found by determining the solubility of individual FAs and their esters that long-chain FAs are less soluble compared to short-chain ones, and saturated FAs are less soluble than monoenoic and dienoic ones of the same chain length; *trans* isomers are less soluble than *cis* isomers, and normal acids are less soluble compared to branched ones [24,35,36]. Thus, the melting point of FAs changes depending on the type and degree of unsaturation, which allows for the separation of SFA and UFA mixture [37]. At a low temperature, mainly SFAs are subject to crystallization, and, to a lesser extent, MUFA, whereas PUFAs, including *n*-3 FAs, stay in the liquid phase.

In the previous study of Bodkowski et al. [25], the authors recommended acetone as a solvent, oil-to-solvent ratio of 1:2.5, and freezing temperature of −70 °C in order to optimize the low-temperature crystallization (LTC) process for fish oil from sprat-herring waste, including an increase in the concentration of *n*-3 FAs, and such conditions were used in this study.

In our study, the content of UFAs in fish oil increased as a result of the LTC process compared to raw fish oil. The largest increase was recorded for DHA, EDA, EPA, and DPA. The increase in UFA content was accompanied by a decrease in SFAs as well as MUFAs.

Omega-3 LC-PUFAs are not stable chemically; thus, when affected by physicochemical and biological factors, marine oils are subject to rapid oxidation during storage to a complex chemical soup of lipid peroxides, secondary oxidation products, and decreasing concentrations of non-oxidized FAs [38]. Omega-3 LC-PUFAs are highly susceptible to the oxidation process because of the large number of double bonds and their position within the FA chain. The oxidation process results in the formation of peroxides as well as aldehydes and ketones that give an unpleasant taste and smell and act as catalysts for further oxidation. The process may also result in the formation of toxic substances and unfavorable changes in the configuration of FAs from *cis* to *trans* [38,39]. Therefore, the primary oxidation products of *n*-3 LC-PUFAs are chemically different from non-oxidized ones and may exhibit different biological properties [38]. In the previous study of Patkowska-Sokoła et al. [26], α-tocopherol in the amount of 0.2% was used as an antioxidant for mackerel oil enriched with in LC-PUFA acids, mainly EPA and DHA, by a urea complexing method. After 4 weeks (cooling conditions, dark closed glass bottles), the decrease in EPA and DHA content did not exceed 2–4% and the peroxide and anisidine values increased slightly. In this study, to protect LC-PUFAs from oxidation processes, α-tocopherol was used as an antioxidant in an analogous amount, i.e., 200 mg/L LTC-FO, and was stored under analogous conditions until application in the study (max. 2 weeks).

Because of the oily form of LTC-FO, it was sprayed on a mineral carrier Humokarbowit in order to facilitate its use in cows’ feed. Humokarbowit consists of natural humus-mineral raw materials like humic acids and their salts, bitumen, lignin, hemicellulose, wax, resins, phytoenzymes, phytohormones, proteins and amino acids, polysaccharides, as well as a rich set of macro- and microelements. Due to its biostimulating and prophylactic properties, it is suitable for poultry, pigs, cattle, and sheep feeding [1,40].

### 4.2. Yield and Chemical Composition of Milk

The elaborated feed additive, i.e., fish oil after a process of low-temperature crystallization (LTC-FO), supplementation to TMR had no effect on the milk production level of the cows. Earlier reports have shown that dietary fish oil (FO) supplements have no effect [41] or increase/decrease milk yield dependent on the amount [14,42,43] and source [44]. In the study of Donovan et al. [42], an increase in dietary fish oil from 0 to 1% caused an increase in milk yield (31.7, 34.2, 32.3, and 27.4 kg/d), while a 1 to 3% increase in dietary fish oil level caused its linear decrease. In the research of Kairenius et al. [14], 75 and 150 g/d FO addition had no effect, but 300 g/d decreased milk yield. In turn, Kupczyński et al. [17] noted an increase in milk yield with 2% FO addition. LTC-FO also had no effect on the yield and concentration of protein and lactose content of milk. Earlier studies have reported that dietary FO supplements often lower milk protein output or milk protein concentrations [42,44,45]. In the studies of Offer et al. [45] and Loor et al. [46], the addition of fish oil had no effect on milk lactose secretion, whereas in Kairenius et al. [14] and Donovan et al. [42], it decreased, and in Keady et al. [44], it increased. In the present study, however, the yield fat and concentration of fat in milk decreased as a result of using the elaborated feed additive. Supplements of FO, especially in non-protected form, usually cause a decrease in milk fat synthesis in lactating cows [47,48,49], a response associated with the effects of FA in FO on ruminal lipid metabolism [46]. In the study of Kairenius et al. [14], FO addition in the amounts of 70, 150, and 300 g/d caused a decrease in fat concentration in cow’s milk (5.6, 19.9, and 30.1%) and fat yield (5.2, 15.1, and 40.6%). In the experiment of Donovan et al. [42], fat concentration (2.97, 2.79, 2.37, 2.3%) and fat yield (0.93, 0.92, 0.67, 0.66 kg/d) in cow’s milk decreased linearly from control vs. 1, 2, to 3% as dietary FO. In turn, in the study of Whitlock et al. [48], the addition of 0.33, 0.67, and 1% of fish oil decreased the fat concentration from 4.2 to 3.81, 3.80, and 4.03%. The addition of 60 to 70 g of fishmeal oil in the cow’s diet would be enough to inhibit milk fat synthesis [50].

Certain dietary conditions in dairy cows, e.g., diets supplemented with PUFA of marine origin (mainly fish oils and algae) cause milk fat depression (MFD) [51,52]. Several theories have been proposed in order to explain diet-induced MFD. For diets including marine supplements, an extension of the biohydrogenation (BH) theory was proposed which attributes the decrease in milk fat level to ruminal lipid metabolism changes leading to the increased formation of specific BH intermediates, inhibiting milk fat synthesis [53,54,55]. In the study of Ahnadi et al. [56], dairy cows’ diet supplementation with protected fish oil caused a decrease in the abundance of lipogenic enzymes mRNA (acetyl-CoA carboxylase (ACC), stearoyl-CoA desaturase (SCD), fatty acid synthase (FASN)) in the mammary gland. In turn, unprotected fish oil supplementation slightly decreased the mRNA levels of these enzymes but markedly reduced the level of lipoprotein lipase mRNA. These results demonstrated that fish oil was responsible for milk fat decrease due to the inhibition of mammary lipogenic enzymes’ gene expression.

In the study of Kadegowda et al. [57], *trans*-10 18:1 have been shown to induce changes in lipogenic gene expression, characterized as a downregulation of FASN, SCD, and sterol regulatory element binding transcription factor 1(SREBF1). Postruminal infusion of a mixture of 18:1 methyl esters supplying *trans*-10 18:1 over a 5-d period resulted in an approximately 20% decrease in milk fat output [58]. Increase in milk *trans*-10 18:1 content is a characteristic feature of MFD induced by a diet [53,55]. Milk fat secretion decrease was also related to an increase in milk fat concentration of *cis*-11 18:1 [50]. The investigation of Burns et al. [59] showed that *cis*-11 18:1 lowered lipogenesis and FASN expression during incubation with bovine adipocyte cultures. In our study, as a result of LTC-FO supplementation, the share of both *trans*-10 C18:1 and *cis*-11 C18:1 in milk fat increased significantly. The addition of LTC-FO also resulted in an increase in milk fat *trans*-11 C18:1 (TVA) content, which probably also inhibited delta-9 desaturase activity, which is responsible for fat synthesis in the mammary gland [60].

During FO-induced MFD, milk fat synthesis decrease is accompanied by lowered proportions of stearic (18:0) and oleic acid (OA, *cis*-9 18:1) in milk fat and higher *trans* 18:1 isomer levels [14,42,43]. Milk fat triacylglycerol synthesis has a stringent requirement for oleic acid synthesized from stearic acid for milk fat fluidity and efficient ejection of fat from the mammary glands [61]. Decreases in the availability of 18:0 together with an increase in the level of *trans* 18:1 isomers that are not substrates for SCD may also contribute to FO-induced MFD [46,50,55]. Probably, the reduction of milk fat in LTC-FO supplementation could be attributed to the decreased proportion of 18:0 and cis-9 18:1 and increased trans 18:1 isomers.

Some studies have demonstrated that CLA isomers may cause the inhibition of milk fat secretion in dairy cows [62,63]. *Trans*-10,*cis*-12 CLA is the only intermediate unequivocally proven to inhibit milk fat synthesis [53,64]. In our experiment, supplementation with LTC-FO caused a slight increase in the content of this isomer after 30 days, while in the study of Kairenius et al. [14], FO in amounts of 75 and 150 g/d caused a decrease in the content of this isomer in cow’s milk fat. With some evidence [64,65], *cis*-10,*trans*-12 and *trans*-9,*cis*-11 CLA may also lower milk fat concentration and yield in lactating cows. Both in our study (after 14 and 30 d) and in that of Kairenius et al. [14], the addition of FO caused an increase in *trans*-9,*cis*-11 CLA content in milk fat.

### 4.3. Fatty Acid Profile of Milk

The concentrations of FAs in milk fat were changed by LTC-FO addition. For example, the shares of short- and medium-chain fatty acids (C4–C16:0) decreased, long-chain fatty acids increased (C > 20), and the level of milk fat saturation was lower.

The synthesis of milk fat depends on two main FA sources, i.e., FA de novo synthesis in the mammary gland and preformed FA transfer from blood TGs. Short- and medium-chain FAs (C4–C14) and half of C16 FAs are synthesized *de novo*. Delivered with diet *n*-3 PUFA and the products of their ruminal BH strongly inhibit fat synthesis in the mammary gland [66,67]. Supplements of fish oil caused a decrease in the relative proportions of 4- to 16-carbon FAs in milk [14,52], which can be explained by the inhibitory effects of an increase in long-chain FA availability on acetyl-CoA carboxylase 1 (ACACA) and FASN activity and de novo synthesis of FAs in secretory cells of the mammary gland [68]. Earlier studies on lactating cows have shown that FO results in a dose-dependent decrease in the proportions of FAs synthesized *de novo* [42,43,44] and lowers mammary mRNA abundance [56]. In our study, the concentrations of particular SCFAs, including C4, C6, C8, and C10, and MCFAs, including C12, C14, and C16, decreased significantly in the response of cow’s diet LTC-FO supplementation. The addition of LTC-FO also resulted in a decrease in milk fat content of stearic acid (C18:0) and oleic acid (*cis*-9 C18:1). A decrease in 18:0 and *cis*-9 18:1 levels is a natural feature of the changes in milk fat composition in relation to FO supplementation [42,43,45], due to the lowered availability of 18:0 for direct incorporation into milk fat or for endogenous synthesis of *cis*-9 18:1 in the mammary gland. As observed in our study, a decrease in the share of laurynic, myristic, palmitic, and stearic acids is important because these FAs play an important role in the formation of the level of total cholesterol and its LDL fractions in blood and as dietary factors may be considered the cause of cardiovascular disease formation [13,31].

On the other hand, LTC-FO supplementation caused an increase the content of *trans* isomers C18:1 in milk fat. In the study of Shingfield et al. [52], fish oil increased milk *trans* FA concentrations several-fold, which can be attributed to the incomplete ruminal BH of unsaturated 16- to 22-carbon FAs. Studies in vitro have demonstrated that 20:5 (EPA, *n*-3) and 22:6 (DHA, *n*-3) inhibit the reduction of 18-carbon unsaturated FAs to 18:0, causing an accumulation of *trans* 18:1 intermediates [69,70]. Earlier studies have also reported that the increase in milk total *trans* FA content related to FO was associated with the specific enrichment of *trans* 18:1 (Δ8–12) and *trans* 18:2 (Δ9,11; Δ9,12; Δ11,15) isomers [46,71]. Similarly to our study, an increase in the content of *trans* C18:1 isomers of configuration *trans*-9, *trans*-10, and *trans*-11 (TVA) was also found in the studies of Kairenius et al. [14] and Donovan et al. [42].

Consistent with earlier reports, FO also increased the content of *cis*-9,*trans*-11 CLA in milk [42,45,71], which can be explained by an increased supply of *trans*-11 18:1 [52] for endogenous *cis*-9,*trans*-11 CLA synthesis by delta-9 desaturase in the mammary gland [72]. In our research, the content of *cis*-9,*trans*-11 CLA (RA) isomer increased 3.7 and 4.1-fold, respectively, after 14 and 30 days of experiment, compared to the CTRL group. An increase in the RA content in milk fat was also found by Kairenius et al. [14] (0.61 vs. 1.03, 2.15, and 2.07 g/100 g of total FAs; control vs. 75, 150, and 300 g/d of FO) and Donovan et al. [42] (0.60, 1.58, 2.23, 1.9 g/100 g of total FAs; 0, 1, 2, and 3% FO of ration DM).

Supplements of FO elevated milk 20:5 (EPA, *n*-3), 22:5 (DPA, *n*-3), and 22:6 (DHA, *n*-3) concentrations in a dose-dependent manner [43,73,74]. The rather low apparent transfer of EPA and DHA from the diet into the milk of lactating cows reflects the extensive BH of these FAs in the rumen [46,52,71]. Following absorption, these FAs are incorporated into cholesterol esters and phospholipids that have a low affinity for lipoprotein lipase in the mammary endothelium [45]. In our experiment, the addition of LTC-FO caused an increase in milk fat EPA, DPA, and DHA. In the research of Kupczyński et al. [17], the addition of 2% fish oil resulted in an increase in EPA and DHA content. Kairenius et al. [14] found an increase in EPA, DHA, and DPA content at the highest amount of 300 g/d FO, while 75 and 150 g/d FO did not influence the content of these FAs. In turn, the addition of FO in doses of 1, 2, and 3% of DM caused an increase in EPA content, while the increase in DHA and DPA was not significant [42].

As a result of LTC-FO supplementation, also the content of C18:3 (ALA, *n*-3), C18:3 (GLA, *n*-6), C18:4 (SDA, *n*-3), C20:1, C20:2 (EDA, *n*-6), and C20:3 (DGLA, *n*-6) in milk fat increased, which is confirmed by the results of other studies [14,17].

Feed additive LTC-FO also modified the profiles of FA groups with different levels of saturation and various carbon chain lengths. In milk fat, the content of SFAs decreased, including SCFAs and MCFAs, and UFAs increased, including MUFAs, PUFAs, and LC-PUFAs. A decrease in SFA and SCFA content and increase in UFAs and LCFAs in cow’s milk were noted by Donovan et al. [42] and Kupczyński et al. [17]. In turn, Kairenius et al. [14] noted a decrease in SFAs and an increase in MUFAs and PUFAs.

As a result of LTC-FO supplementation, the content of *n*-3 PUFAs in milk fat increased, as it did in the studies of Donovan et al. [42], Kupczyński et al. [17], and Withlock et al. [48].

The desaturase index (DI), which is calculated on the basis of the pairs of FAs which represent the product–substrate for delta-9 desaturase, is a kind of a proxy for this enzyme activity in the mammary gland [53]. In the case of MFD resulting from dietary reasons, the milk fat desaturase index may be altered, and FO leads to a decrease in milk fat and a shift in DI. Kairenius et al. [14] demonstrated an increase in the ratios of 16:1/C16 and C18:1/18:0 and decrease in RA/TVA following FO administration. In the studies of Kupczyński et al. [17] and AbuGhazaleh et al. [75], FO supplementation had no effect on the CLA/TVA ratio. In the present study, the addition of LTC-FO resulted in a decrease in RA/TVA ratio, increase in C16:1/C16 and C14:1/C14 ratios, and had no effect on C18:1/18:0.

Considering the negative role of C12:0, C14:0, C16:0, and C18:0, the atherogenic (AI) and thrombogenic (TI) indices were proposed by Ulbricht and Southgate [31]. In our study, the value of AI and TI decreased as a result of LTC-FO addition. In the study of Donovan et al. [42], the addition of FO caused a decrease in AI value.

## 5. Conclusions

Dietary supplementation of dairy cows’ feed with 1% of LTC-FO caused a reduction in milk fat content, and this phenomenon had been confirmed in previous studies, which demonstrated that FO addition to the diet reduced the content of milk fat in lactating animals. Lowered milk fat secretion was associated with a lower share of short-chain FAs (C4–10) as well as medium-chain ones (C12–16) (i.e., laurynic, myristic, and palmitic acids), which is consistent with the inhibitory effect of the applied dietary supplement on de novo FA synthesis. In turn, the level of biologically active FAs with health promoting properties (i.e., CLA, TVA, ALA, EPA, DPA, DHA) significantly increased in milk fat following LTC-FO supplementation. An increase in the content of these acids was associated with a higher supply of n-3 LC-PUFA in the ration of cows receiving the elaborated additive and their influence on the processes taking place in the rumen. The addition of LTC-FO also favorably modified the ratios of SFA/UFA and *n*-6/*n*-3 in milk fat and reduced the values of atherogenic and thrombogenic indices. This study demonstrated that fish oil after the process of low-temperature crystallization, characterized by high levels of *n*-3 PUFAs, can be successfully applied in dairy cows’ feed in order to change the nutritional quality and associated health value of milk, which may be beneficial also for human health.

## Figures and Tables

**Table 1:** Components and nutritive value of cow diets.

Specification	Treatment ^1^	
	CTRL	EXP
Components, % of DM		
Corn silage	38.5	38.5
Grass silage	25	25
Fresh spent grain	7	7
Wet beet pulp	6.5	6.5
Rapeseed meal	6	5
Soybean meal	3.5	3.5
Second-cut hay	4.5	4.5
Complete mixture	7	7
Humokarbowit ^2^	1.3	1.3
Fish oil after low-temperature crystallization	-	1
Calcium bicarbonate	0.25	0.25
Vitamins and minerals ^3^	0.45	0.45
Nutritive value of ration		
DM (kg)	24.9	24.7
NE_L_^4^ (Mcal/kg)	1.53	1.57
Crude protein (% DM)	15.2	15.1
Crude fat (% DM)	3.07	4.09
NDF ^5^ (% DM)	35.5	35.5
ADF ^6^ (% DM)	25.2	25.2
Calcium (% DM)	0.74	0.72
Phosphorus (% DM)	0.38	0.37
DMI ^7^ (kg)	23.7	22.8

^1^ Treatments: CTRL, control group; EXP, experimental group supplemented with fish oil after low-temperature crystallization (LTC-FO). ^2^ Humic-mineral preparation (PHW Tronina, Raków, Poland). ^3^ Fatromix BoW3:1 (Fatro Polska Ltd., Kobierzyce, Poland). ^4^ NE_L_, net energy for lactation. ^5^ NDF, neutral detergent fiber. ^6^ ADF, acid detergent fiber. ^7^ DMI, dry matter intake.

**Table 2:** Main fatty acids in raw sprat-herring oil (FO) and after process of low-temperature crystallization (LTC-FO) (mean ± SD).

Fatty Acids (g/100 g of Fat)	Fish Oil	
	Raw Form (FO)	After Process of Low-Temperature Crystallization (LTC-FO)
Saturated fatty acids (SFAs)		
C14:0	6.34 ± 1.16 ^A^	2.72 ± 0.42 ^B^
C16:0	15.43 ± 2.12 ^A^	2.67 ± 0.48 ^B^
C18:0	2.52 ± 0.39 ^A^	0.68 ± 0.12 ^B^
Monounsaturated fatty acids (MUFAs)		
C16:1n-7	7.53 ± 1.33 ^A^	3.36 ± 0.56 ^B^
C17:1n-7	2.48 ± 0.44 ^a^	1.62 ± 0.52 ^b^
C18:1n-9	24.33 ± 3.31	21.24 ± 2.89
C20:1n-9	2.45 ± 0.69	3.05 ± 0.54
Polyunsaturated fatty acids (PUFAs)		
C18:2n-6	4.26 ± 1.26	4.36 ± 1.35
C18:3n-3	3.83 ± 0.76	3.93 ± 0.48
C18:4n-3	2.23 ± 0.69 ^a^	2.78 ± 0.54 ^b^
C20:2n-6	1.14 ± 0.28 ^A^	2.38 ± 0.42 ^B^
C20:5n-3	6.46 ± 1.45 ^A^	12.26 ± 1.78 ^B^
C22:5n-3	1.82 ± 0.35 ^A^	3.26 ± 0.40 ^B^
C22:6n-3	14.48 ± 2.41 ^A^	30.57 ± 3.32 ^B^
SFAs	25.34 ± 2.98 ^A^	6.57 ± 0.38 ^B^
UFAs ^1^	73.78 ± 2.56 ^A^	92.27 ± 3.02 ^B^
MUFAs	38.16 ± 2.77 ^A^	30.55 ± 2.56 ^B^
PUFAs	35.62 ± 2.32 ^A^	61.72 ± 3.13 ^B^

^a–b^ Different superscripts indicate statistical differences between mean values in the rows at *p* < 0.05. ^A–B^ Different superscripts indicate statistical differences between mean values in the rows at *p* < 0.01. ^1^ UFAs, unsaturated fatty acids MUFAs + PUFAs.

**Table 3:** Selected parameters of milk from control (CTRL) and experimental (EXP) cows at two times of sample collection (14 and 30 d).

Item	CTRL ^1^		EXP ^2^		SEM ^3^	Effect ^4^		
	14 d	30 d	14 d	30 d		D	T	D × T
Yield (kg/head/day)								
Milk	31.35	30.98	32.12	32.42	1.76	NS	NS	NS
Fat	1.39 ^A^	1.38 ^A^	1.03 ^B^	1.07 ^B^	0.11	**	NS	NS
Protein	1.04	1.02	1.02	1.04	0.02	NS	NS	NS
Lactose	1.47	1.46	1.53	1.52	0.04	NS	NS	NS
Concentration (g/kg)								
Total solids	125.23 ^a^	124.78 ^a^	112.73 ^b^	113.85 ^b^	3.23	*	NS	NS
Fat	44.22 ^A^	44.64 ^A^	32.15 ^B^	33.11 ^B^	3.76	**	NS	NS
Protein	33.26	33.02	31.71	32.23	1.58	NS	NS	NS
Lactose	46.85	47.28	47.54	47.10	0.49	NS	NS	NS
SCC ^5^, log10 cells/mL	2.65	2.34	2.58	2.67	0.38	NS	NS	NS

^a-b^ Different superscripts indicate statistical differences between mean values in the rows at *p* < 0.05. ^A–B^ Different superscripts indicate statistical differences between mean values in the rows at *p* < 0.01.^1^ CTRL, control group with no additional oil; ^2^ EXP, experimental group supplemented with the addition of 1% of DM of fish oil after low-temperature crystallization (LTC-FO); ^3^ SEM, standard error of the mean; ^4^ effects due to diet (D), time (T), and their interaction (D × T); * *p* < 0.05; ** *p* < 0.01; NS, not significant; ^5^ SCC, somatic cell count.

**Table 4:** Mean values of fatty acid (FA) content in milk from control (CTRL) and experimental (EXP) cows at two times of sample collection (14 and 30 d).

Fatty Acids, g/100 g of Total FAs	CTRL ^1^		EXP ^2^		SEM ^3^	Effect ^4^		
	14 d	30 d	14 d	30 d		D	T	D × T
C4:0 (BA)	2.983 ^A^	3.298 ^A^	2.467 ^B^	2.511 ^B^	0.185	**	NS	NS
C6:0	1.856 ^A^	1.796 ^A^	1.378 ^B^	1.402 ^B^	0.117	**	NS	NS
C8:0	1.544 ^A^	1.692 ^A^	1.195 ^B^	1.035 ^B^	0.078	**	NS	NS
C10:0	2.475 ^A^	2.756 ^A^	1.788 ^B^	1.735 ^B^	0.182	**	NS	NS
C12:0	3.387 ^A^	3.439 ^A^	2.674 ^Ba^	2.870 ^Bb^	0.223	**	*	NS
C14:0	10.574 ^a^	10.145 ^ab^	9.746 ^bc^	9.373 ^c^	0.298	*	NS	NS
C14:1	0.855	0.798	0.835	0.901	0.054	NS	NS	NS
C16:0	29.640 ^a^	29.246 ^ab^	27.591 ^bc^	27.230 ^c^	0.490	*	NS	NS
C16:1	0.895 ^A^	0.940 ^A^	1.942 ^B^	2.006 ^B^	0.062	**	NS	*
C18:0	14.370 ^a^	15.064 ^A^	12.811 ^Bb^	12.679 ^Bb^	0.321	**	NS	NS
C18:1 (OA)	23.334 ^a^	22.940	20.532 ^b^	21.18	1.012	*	NS	NS
C18:1 t9	0.166 ^A^	0.153 ^A^	0.288 ^B^	0.267 ^B^	0.036	**	NS	NS
C18:1 t10	0.280 ^A^	0.230 ^A^	0.640 ^B^	0.558 ^C^	0.042	**	**	*
C18:1 c11	0.337 ^A^	0.348 ^A^	0.816 ^B^	0.844 ^B^	0.050	**	NS	NS
C18:1 t11 (TVA)	1.270 ^A^	1.229 ^A^	5.411 ^B^	5.866 ^B^	0.365	**	NS	NS
C18:2 (LA)	2.372	2.479	2.223	2.135	0.139	NS	NS	NS
C18:2 c9,t11 (CLA)	0.645 ^A^	0.630 ^A^	2.370 ^B^	2.584 ^B^	0.119	**	*	NS
C18:2 t9,c11 (CLA)	0.012 ^A^	0.010 ^A^	0.036 ^Ba^	0.031 ^Ba^	0.008	**	*	NS
C18:2 t10,c12 (CLA)	0.006 ^A^	0.007 ^a^	0.008 ^B^	0.009 ^Bb^	0.002	*	NS	NS
C18:2 t11,c13 (CLA)	0.022 ^A^	0.019 ^A^	0.014 ^B^	0.010 ^C^	0.005	**	**	*
C18:3 (ALA)	0.573 ^A^	0.526 ^A^	0.778 ^B^	0.944 ^C^	0.143	**	**	**
C18:3 (GLA)	0.018 ^A^	0.015 ^A^	0.036 ^B^	0.034 ^B^	0.011	**	NS	*
C18:4 (SDA)	0.006 ^A^	0.005 ^A^	0.022 ^B^	0.024 ^B^	0.004	**	NS	NS
C20:0	0.156	0.143	0.133	0.120	0.033	NS	NS	NS
C20:1	0.141 ^A^	0.137 ^A^	0.620 ^B^	1.038 ^C^	0.019	**	**	**
C20:2(EDA)	0.058 ^A^	0.056 ^A^	0.229 ^B^	0.254 ^B^	0.022	**	NS	NS
C20:3 (DGLA)	0.037 ^A^	0.041 ^A^	0.067 ^Ba^	0.085 ^Bb^	0.018	**	*	NS
C20:4 (AA)	0.053	0.047	0.055	0.057	0.008	NS	NS	NS
20:5 (EPA)	0.032 ^A^	0.034 ^A^	0.078 ^Ba^	0.092 ^Bb^	0.011	**	*	NS
C22:0	0.048	0.051	0.052	0.046	0.006	NS	NS	NS
C22:1	0.035 ^a^	0.032 ^a^	0.044	0.052 ^b^	0.011	*	NS	NS
C22:2	0.042 ^a^	0.037	0.034 ^b^	0.038	0.006	*	NS	NS
C22:5 (DPA)	0.047 ^A^	0.044 ^A^	0.116 ^B^	0.122 ^B^	0.008	**	NS	NS
C22:6 (DHA)	0.021 ^A^	0.019 ^A^	0.073 ^Ba^	0.085 ^Bb^	0.011	**	*	NS
C24:0	0.016	0.014	0.014	0.015	0.003	NS	NS	NS
Unidentified	1.695	1.580	1.924	1.805	0.368	NS	NS	NS

^a-c^ Different superscripts indicate statistical differences between mean values in the rows at *p* < 0.05. ^A–C^ Different superscripts indicate statistical differences between mean values in the rows at *p* < 0.01. ^1^ CTRL, control group with no additional oil; ^2^ EXP, experimental group supplemented with the addition of 1% of DM of fish oil after low-temperature crystallization (LTC-FO); ^3^ SEM, standard error of the mean; ^4^ effects due to diet (D), time (T), and their interaction (D x T); * *p* < 0.05; ** *p* < 0.01; NS, not significant; BA—butyric acid; OA—oleic acid; TVA—trans-vaccenic acid; LA—linoleic acid; CLA—conjugated linoleic acid; ALA—α-linolenic acid; GLA—γ-linoleic acid; SDA—stearidonic acid; EDA—eicosadienoic acid; DGLA—dihomo-γ-linolenic acid; AA—arachidonic acid; EPA—eicosapentaenoic acid, DPA—docosapentaenoic acid; DHA—docosahexaenoic acid.

**Table 5:** Mean values of FA groups, *n*-3, SFA/UFA, *n*-6/*n*-3, delta-9 desaturase index, and atherogenic and thrombogenic indices in milk from control (CTRL) and experimental (EXP) cows at two times of sample collection (14 and 30 d).

Item	CTRL ^1^		EXP ^2^		SEM ^3^	Effect ^4^		
	14 d	30 d	14 d	30 d		D	T	D x T
SFA ^5^	67.05 ^A^	67.64 ^A^	60.85 ^B^	59.02 ^B^	1.738	**	NS	NS
UFA ^6^	31.26 ^A^	30.78 ^A^	37.26 ^Ba^	39.21 ^Bb^	1.121	**	*	NS
MUFA ^7^	27.31 ^A^	26.81 ^A^	31.13 ^B^	32.72 ^B^	0.764	**	NS	NS
PUFA ^8^	3.94 ^A^	3.97 ^A^	6.13 ^Ba^	6.49 ^Bb^	0.258	**	*	NS
SCFA ^9^	8.86 ^A^	9.54 ^A^	6.82 ^B^	6.68 ^B^	0.347	**	NS	NS
MCFA ^10^	45.35 ^a^	44.57 ^a^	43.79	42.38 ^b^	0.848	*	NS	NS
LCFA ^11^	44.10 ^Aa^	44.31 ^A^	47.49 ^b^	49.16 ^Bc^	1.173	**	NS	NS
LC-PUFA ^12^	0.29 ^A^	0.28 ^A^	0.64 ^B^	0.72 ^C^	0.022	**	**	**
SFA/UFA	2.14 ^A^	2.20 ^A^	1.63 ^B^	1.51 ^B^	0.037	**	NS	NS
Σ CLA ^13^	0.68 ^A^	0.67 ^A^	2.43 ^B^	2.63 ^B^	0.173	**	NS	NS
Σ EPA + DHA^14^	0.05 ^A^	0.05 ^A^	0.15 ^Ba^	0.18 ^Bb^	0.017	**	*	NS
FFA ^15^	31.37 ^Aa^	30.62 ^Aa^	34.15 ^b^	35.62 ^B^	0.266	**	NS	NS
n-3 ^16^	0.68 ^A^	0.63 ^A^	1.06 ^Ba^	1.31 ^Bb^	0.115	**	*	*
n-6/n-3	3.74 ^A^	4.19 ^A^	2.53 ^B^	1.99 ^C^	0.056	**	**	**
DI ^17^								
C14:1/(C14:0 + C14:1)	0.07 ^a^	0.07 ^a^	0.08	0.09 ^b^	0.008	*	NS	NS
C16:1/(C16:0 + C16:1)	0.03 ^A^	0.03 ^A^	0.06 ^B^	0.07 ^B^	0.006	**	NS	NS
C18:1/(C18:0 + C18:1)	0.62	0.60	0.61	0.62	0.033	NS	NS	NS
cis-9,trans-11 CLA / trans-11 C18:1	0.34 ^a^	0.34 ^a^	0.30 ^b^	0.31 ^b^	0.016	*	NS	NS
AI ^18^	2.41 ^A^	2.38 ^A^	1.88 ^B^	1.71 ^B^	0.046	**	NS	NS
TI ^19^	2.75 ^A^	2.83 ^A^	2.16 ^Ba^	1.92 ^Bb^	0.058	**	*	NS

^a-b^ Different superscripts indicate statistical differences between mean values in the rows at *p* < 0.05. ^A–C^ Different superscripts indicate statistical differences between mean values in the rows at *p* < 0.01. ^1^ CTRL, control group with no additional oil; ^2^ EXP, experimental group supplemented with the addition of 1% of DM of fish oil after low-temperature crystallization (LTC-FO); ^3^ SEM, standard error of the mean; ^4^ effects due to diet (D), time (T), and their interaction (D x T); * *p* < 0.05; ** *p* < 0.01; NS, not significant; ^5^ SFAs, saturated fatty acids; ^6^ UFAs, unsaturated fatty acids MUFA + PUFA; ^7^ MUFAs, monounsaturated fatty acids; ^8^ PUFAs, polyunsaturated fatty acids; ^9^ SCFAs, short-chain fatty acids (FAs with C4–10); ^10^ MCFAs, medium-chain fatty acids (FAs with C12–16:0); ^11^ LCFAs, long-chain fatty acids (FA ≥ C17:0); ^12^ LC-PUFAs, long-chain polyunsaturated fatty acids (PUFA with twenty or more atoms of carbon), ^13^ Σ CLA, Σ C18:2 *cis*-9,*trans*-11; *trans*-9,*cis*-11; *trans*-10,*cis*-12; *trans*-11,*cis*-13; ^14^ Σ EPA + DHA, Σ eicosapentaenoic acid (C20:5, EPA) + docosahexaenoic acid (C22:6, DHA), ^15^ FFAs, functional fatty acid Σ C4:0 (BA), C18:1 (OA), C18:1 (TVA), C18:2 (LA), isomers CLA, C18:3 (ALA), C20:4 (AA), C20:5 (EPA), C22:5 (DPA), C22:6 (DHA); ^16^ *n*-3, Σ C18:3 *cis*-9,12,15; C18:4 *cis*-6,9,12,15; 20:5 *cis*-5,8,11,14,17; C22:5 *cis*-7,10,13,16,19; 22:6 *cis*-4,7,10,13,16,19; ^17^ DI, desaturase index for delta-9 desaturase; ^18^ AI, atherogenic index calculated from (C12:0 + (4 × C14:0) + C16:0)/(MUFAs + PUFAs) [31]; ^19^ TI, thrombogenic index calculated from (12:0 + 16:0 + 18:0)/[(0.5 × MUFA) + (0.5 × n-6 PUFA) + (3 × n-3 PUFA)+ (n-3 PUFA/n-6 PUFA)] [31].
